# Supplementary material for: Bound to the group and blinded by the leader: ideological leader–follower dynamics in a trust economic game
Source: R Soc Open Sci. 2019 Sep 25;6(9):182023. doi: 10.1098/rsos.182023 (PMC6774964; doi:10.1098/rsos.182023)
Supplement: Electronic supplementary material for the paper "Bound to the group and blinded by the leader: ideological leader-follower dynamics in a trust economic game" [file rsos182023supp1.pdf]

SUPPORTING INFORMATION
Bound to the group and blinded by the leader: Ideological leader-follower dynamics in a trust economic game

Biljana Gjoneska[1,2,3], Marco Tullio Liuzza[2,3,4], Giuseppina Porciello[2,3], Gian Vittorio Caprara[2] and Salvatore M. Aglioti[3,5]

[1]Academy of Sciences and Arts of North Macedonia
[2]Department of Psychology, "Sapienza" University of Rome, Italy
[3]IRCCS Santa Lucia Foundation, Rome, Italy
[4]Department of Surgical and Medical Sciences, "Magna Graecia" University of Catanzaro, Italy
[5]Department of Psychology, Sapienza University of Rome & CLNS@SAPIENZA at the Italian Institute of Technology, Rome, Italy

CONTENTS

Methods: Experimental Design
Results: Indicators of Trust
Results: Evaluation of Trustors and Trustees
Appendix: Figures and Tables

1. METHODS: Experimental Design

1.1 Procedure

Each participant was randomly assigned as a trustor to either a trust or a distrust version of the Trust Game (TG) and endowed with 10 euros which were "renewed" in each of the 15 randomly ordered trials. Each trial began with a short fixation cross (1000ms), followed by an image of the trustee and the participant's economic decision (untimed). The process of making an offer was marked by selecting the corresponding number (to the amount displayed in euros), and followed by the calculation of the quadrupled investment. The trustee's decision was revealed after a pause during which the supposed mathematical model computed his behavior (4000ms), with the summary of the final payoff to each player presented at the end of the trial. See also Fig. 1.

In order to increase participants' engagement in the game they were told that one of the rounds will be randomly selected and paid in addition to the standard rate for participation in the experiment. In reality, each player received fixed bonus rate due to the lack of a real mathematical model.

1.2 Concept

The trust is developed when three conditions are met:
- A risky situation without a contract (signifying an uncertain outcome)
- A positive expectation for a beneficial outcome (despite uncertainty)
- A risky action in the form of an economic investment (see Fig. 1)

In the TG "the willingness to bet that another person will reciprocate a risky move at a cost to himself" is said to capture the concept of trust. The amount returned by the trustee captures trustworthiness [1]. When trust occurs both parties must be better off, suggesting that "trust must produce a welfare-gain in order to facilitate exchange"; Inversely, when trust does not occur, the trustor "is worse off than if he had not trusted at all, adding insult to injury" [2]. Hence, there is not only financial but also psychological risk, so the very act of trusting can be seen as an investment. Misplaced trust can lead not only to the loss of the invested goods, but also the psychological damage of having acted naively [3] Therefore, participants have to overcome their aversion to this risk in order to trust – a situation of particular interest in the investigation of leader-follower relations.See also Fig. 1.

2. RESULTS: Indicators of Trust

2.1 Average amount of investment

To explore the overall dynamics of trust, we employed linear mixed effects modeling (LME) with the R package "lme4" [4], fit by maximum likelihood t-tests using Satterthwaite approximations to degrees of freedom. The listed combinations were investigated in greater detail.

2.1.1. Self-reported Ideology as indicator of economic behavior

Full-interaction Model: Specifications

Refer to Tab.1: Step-wise regression analysis of models with increasing complexity of interaction.

Refer to Tab.2: Descriptive statistics of the winning model Trial N° x Group x Trustee x Condition.

2.1.2. Group binding dimension as indicator of economic behavior

Full-interaction Model: Specifications

Refer to Tab.3: Step-wise regression analysis of models with increasing complexity of interaction.

Refer to Tab.4: Descriptive statistics of the winning model Trial N° x Group x Trustee x Condition.

Full-interaction Model: Illustration

Refer to Fig.3: Heat map model of the economic behavior of participants as a function of their scores on the Group-binding dimension.

2.1.3. Social equality dimension as indicator of economic behavior

Full-interaction Model: Specifications

Refer to Tab.5: Step-wise regression analysis of models with increasing complexity of interaction.

Refer to Tab.6: Descriptive statistics of the winning model Trial N° x Group x Trustee x Condition.

2.2 Trend of investment

In order to check the consistency of our main findings (elaborated in the manuscript) we performed further exploratory analysis investigating additional markers of trust. Thus, we analyzed the economic behavior indexed by the trend of investment, a parameter obtained from the correlation between a numerical sequence (1-15, corresponding to the order of trials) and participant's offers in each consecutive trial.

We ran a 2 x 2 x 2 ANOVA with the Group (LW/RW), Condition (trust/distrust) and Trustee (SB/PA) as between-subjects factors, and the Trend of investment as a dependent variable. We found a significant triple interaction F(1,110) = 6.086, $p < .05$ indicating that there were significant differences in the trend of investment between LW/RW groups playing with the trust/distrust versions of Berlusconi. Crucially for our hypothesis, post-hoc tests (Condition x Trustee) showed that LW-participants who played with Berlusconi displayed a clear difference ($p < .001$) in the trends between the two conditions, lowering investments in the distrust condition while increasing in the trust condition (distrust: mean = -0.327, SD = 0.09; trust: mean = 0.274, SD = 0.09). On the other hand, RW-participants did not show a significant difference ($p = .538$) in the trend between the two TG conditions (distrust: mean = -0.042, SD = 0.09; trust: mean = 0.033, SD = 0.09). In addition, both the LW-group (distrust: mean = -0.216, SD = 0.338; trust: mean = 0.159, SD = 0.299) and the RW-group (distrust: mean = -0.327, SD = 0.316; trust: mean = 0.129, SD = 0.287) displayed a significant difference (LW $p < .01$; RW $p < .001$) in the trend of investment between the two versions of the TG with Angela. See also Fig. 2.

3. RESULTS: Evaluation of Trustors and Trustees

3.1 Evaluation of Trustors

For each measure, we performed an independent samples t-test with the Group (LW/RW) as between-subjects factor, and our findings are consistent with previous literature. Refer to Tab. 7 for a detailed account.

Moral Foundations Questionnaire (MFQ) [5]. The MF theory suggests that conservatives routinely score higher on the "socially binding" moral foundations like Loyalty (people should stay faithful to their groups), Authority (leaders and social institutions are essential for creating social order and should be deferred to unless there is a strong reason not to), and Purity (some people, objects, and ideas must be protected from desecration, particularly those binding a group), while they don't differ from liberals in their ratings on the "individualizing" moral foundations. This is due to the fact that liberals embrace the moral foundations which are less linked with groups but more to individuals such as Fairness (concerns for equality, especially regarding marginalized groups) and Harm avoidance [6, 7]. In our study, we confirmed the findings regarding the "socially binding" dimension showing that conservatives clearly score higher on Authority, Loyalty and Purity. As for the "individualizing" dimension we showed that liberals score higher on Fairness/Reciprocity while there was no significant difference for the Harm/Care scores between the groups. Please refer to Tab. 7

Social Value Orientation (SVO) [8, 9]. The SVO model stems from the view that each individual values the manner in which his own outcomes relate to others' outcomes. This can result in four general tendencies: Altruism (maximization of other's outcomes), Cooperation (maximization of joint outcomes), Individualism (maximizing own outcomes), and Competition (maximizing relative advantage to the other). In line with previous findings, the present research demonstrates that conservatives present lower average SVO scores than liberals. Please refer to Tab. 7

Right-Wing Authoritarian (RWA) and Social Dominance Orientation (SDO) [10, 11, 12]. The RWA measure is mainly related to cluster of traits such as conventionalism, aggression, submission to authority and anti-intellectualism, known as "authoritarian personality" [13]. While RWA focuses on submission to in-group authority figures, SDO focuses on dominance over out-groups. That is, "RWA is seen as an intra-group phenomenon, while SDO is considered as an inter-group phenomenon" [14]. Specifically, RWA indicates the level of in-group inequality, whereas SDO indicates the level of the social inequity on the whole. Both surveys help in defining "the group-centrism syndrome" consisting of the following manifestations: conservatism and resistance to change, pressure to uniformity of opinion, perpetuation of group norms and rejection of deviates, in-group favoritism and out-group derogation, as well as encouragement of autocratic authority. Congruent with the postulated hypothesis and findings from our previous studies [15], the data confirms that the two groups (LW and RW) score differently on these questionnaires, namely RW scored higher on both RWA and SDO than LW.Please refer to Tab. 7

Economic System Justification Inventory (ESJI) [16, 17]. The ESJ Theory postulates that existing social arrangements are often legitimized even at the expense of personal or group interests. By assessing the tendency to "legitimize economic inequality" between the members of high and low status groups (i.e., the quality specific for so-called economic conservatism), our data support previous literature and show differences between RW and LW, with higher scores in the latter as opposed the former group. Please refer to Tab. 7

3.2. Evaluation of Trustees

Control Stimulus: Political Orientation. We analyzed the ideological scores attributed to the chosen control stimulus (Piero Angela), by the sample participating in the experiment (RW- and LW-group of students). A 7-point Likert scale was employed in order to qualify Angela's political orientation (1="extremely left"; 2="left"; 3="center-lef"; 4="center-right"; 5="right"; 6="extremely right", 7="apolitical"). The responses marked as "7" were substituted with "0" (signifying no political orientation whatsoever), while the rest were scaled in the range from -2.5 ("extremely left") to 2.5 ("extremely right"). The results show that the sample of participating students, again rated Angela similarly, with an average score of -0.31, suggesting that indeed he was considered as a figure with low political polarization. In addition, there were no statistical differences between the groups (RW/LW) on the political categorization of Angela ($F(119) = 3.32$; $p = .071$). Hence, both had similar perceptions of Angela and neither LW-voters nor RW-voters perceived him as more politically inclined toward the liberal/conservative ideologies. For details see Tab. 8

Control and Experimental Stimulus: Competence, Valence, Dominance, Acquaintance In order to verify the choice of stimuli we also performed a repeated measures ANOVA on the 121 participants who were included in the survey-analysis with the Trustee (SB/PA) as a within-subjects factor, and the Group (RW/LW) as a between-subjects factor. This was done in order to compare the effects of political orientation in the assessment of Berlusconi and Angela on each of the following dimensions separately: Acquaintance, Dominance, Competence, Positive and Negative Valence. For detailed results see Tab. 9.

References

1. Camerer, C. F. *Behavioral Game Theory: Experiments in Strategic Interaction* (Princeton University Press, 2011).
2. Johnson, N. & Mislin, A. A. Trust games: A meta-analysis. *J Econ Psychol* **32**, 865–889 (2011). DOI: 10.1016/j.joep.2011.05.007.
3. Riegelsberger, J., Sasse, M. A. & McCarthy, J. D. The mechanics of trust: A framework for research and design. *Int J Hum Comput. Stud* **62**, 381–422 (2005). DOI: 10.1016/j.ijhcs.2005.01.001.
4. Bates, D., Maechler, M. & Bolker, B. Linear mixed-effects models using S4 classes. R package version 0999375–42. *R: A Lang. Environ. for Stat. Comput.* (2011).
5. Haidt, J. & Joseph, C. Intuitive ethics: how innately prepared intuitions generate culturally variable virtues. *Daedalus* **133**, 55–66 (2004). DOI: 10.1162/0011526042365555.
6. Haidt, J. & Graham, J. When Morality Opposes Justice: Conservatives Have Moral Intuitions that Liberals may not Recognize. *Soc Just Res* **20**, 98–116 (2007). DOI: 10.1007/s11211-007-0034-z.
7. Graham, J., Haidt, J. & Nosek, B. A. Liberals and conservatives rely on different sets of moral foundations. *J Pers Soc Psychol* **96**, 1029–1046 (2009). DOI: 10.1037/a0015141.
8. Murphy, R. O., Ackerman, K. A. & Handgraaf, M. J. J. Measuring social value orientation. *Judgm Decis Mak* **6**, 771–781 (2011). DOI: 10.2139/ssrn.1804189.
9. Murphy, R. O. & Ackermann, K. A. Social value orientation: theoretical and measurement issues in the study of social preferences. *Pers Soc Psychol Rev* **18**, 13–41 (2014). DOI: 10.1177/1088868313501745.
10. Adorno, T. W., Frenkel-Brunswik, E., Levinson, D. J. & Sanford, R. N. *The Authoritarian Personality. A Volume in Studies in Prejudice Series.* (Harper & Brothers, 1950), 1 edn.
11. Altemeyer, R. A. *Right-Wing Authoritarianism* (Univ of Manitoba Pr, Winnipeg, 1981).
12. Sidanius, J. & Pratto, F. *Social Dominance: An Intergroup Theory of Social Hierarchy and Oppression* (Cambridge University Press, Cambridge, 2001).
13. Altemeyer, B. *The Authoritarian Specter* (Harvard University Press, 1996).
14. Zakrisson, I. Construction of a short version of the Right-Wing Authoritarianism (RWA) scale. *Pers Individ. Dif* **39**, 863–872 (2005). DOI: 10.1016/j.paid.2005.02.026.
15. Ponsi, G., Panasiti, M. S., Aglioti, S. M. & Liuzza, M. T. Right-wing authoritarianism and stereotype driven expectations interact in shaping intergroup trust in one-shot vs multiple-round social interactions. *PLoS ONE* **12**, e0190142 (2018). DOI: 10.1371/journal.pone.0190142.
16. Jost, J. T. & Banaji, M. R. The role of stereotyping in system-justification and the production of false consciousness. *Br. J. Soc. Psychol.* **33**, 1–27 (1994). DOI: 10.1111/j.2044-8309.1994.tb01008.x.
17. Jost, J. T. & Thompson, E. P. Group-Based Dominance and Opposition to Equality as Independent Predictors of Self-Esteem, Ethnocentrism, and Social Policy Attitudes among African Americans and European Americans. *J Exp Soc Psychol* **36**, 209–232 (2000). DOI: 10.1006/jesp.1999.1403.

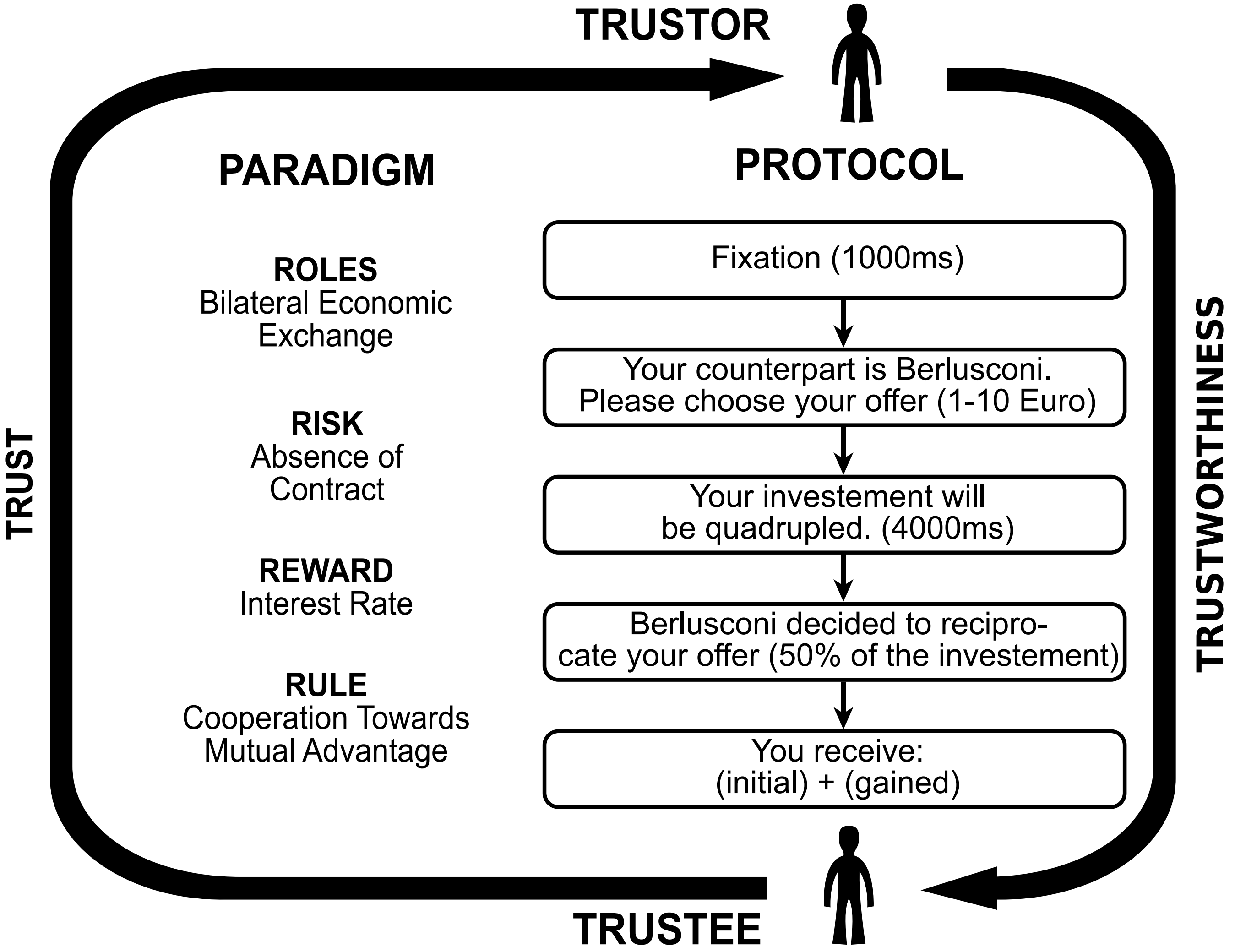

Figure 1. **General Trust Economic Paradigm (left half) and Specific Trust Game Design.** Demonstrated example of game employs Berlusconi as a Trustee (right half)

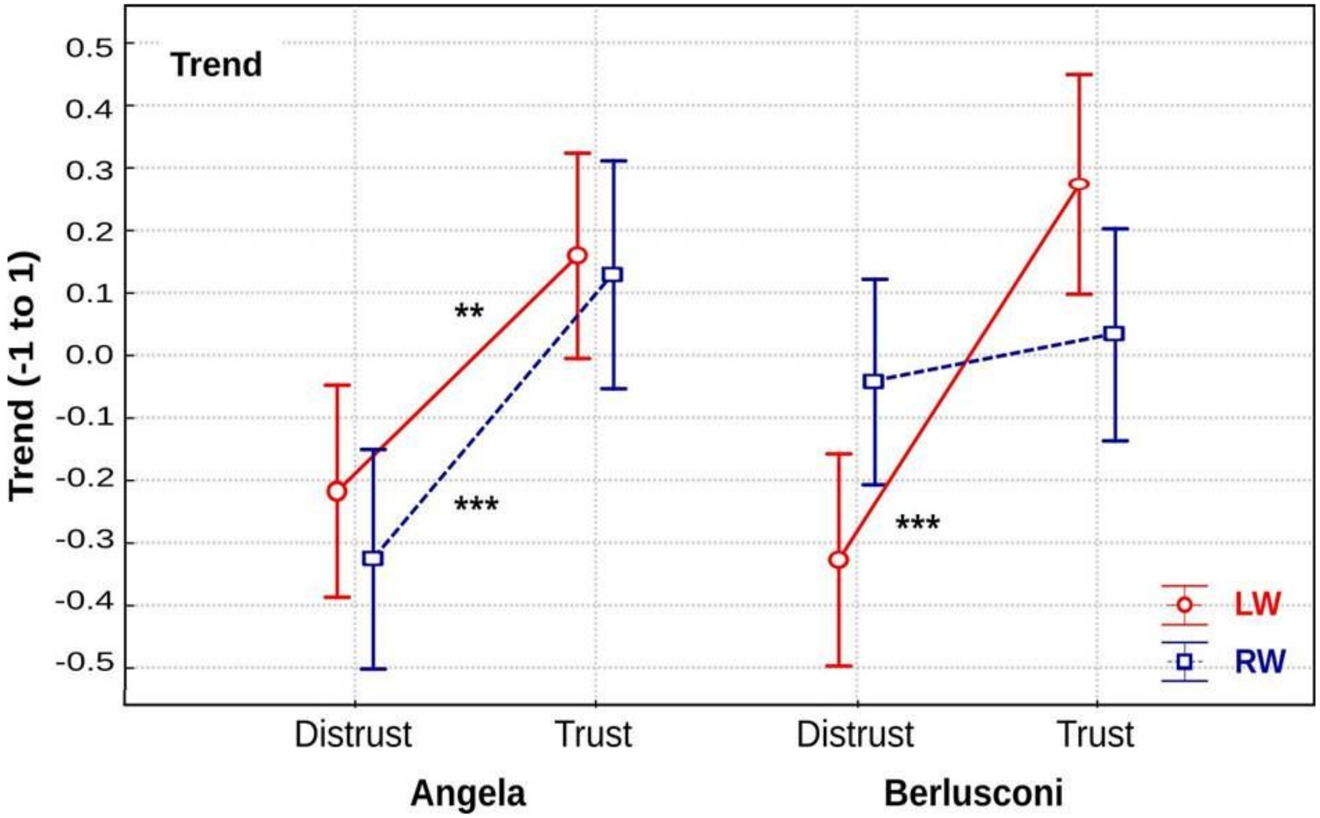

Figure 2. **Trend of investment plotted as function of the Group (LW/RW), TG Condition (trust/distrust) and Trustee (SB/PA)** ** $p < .01$ *** $p < .001$

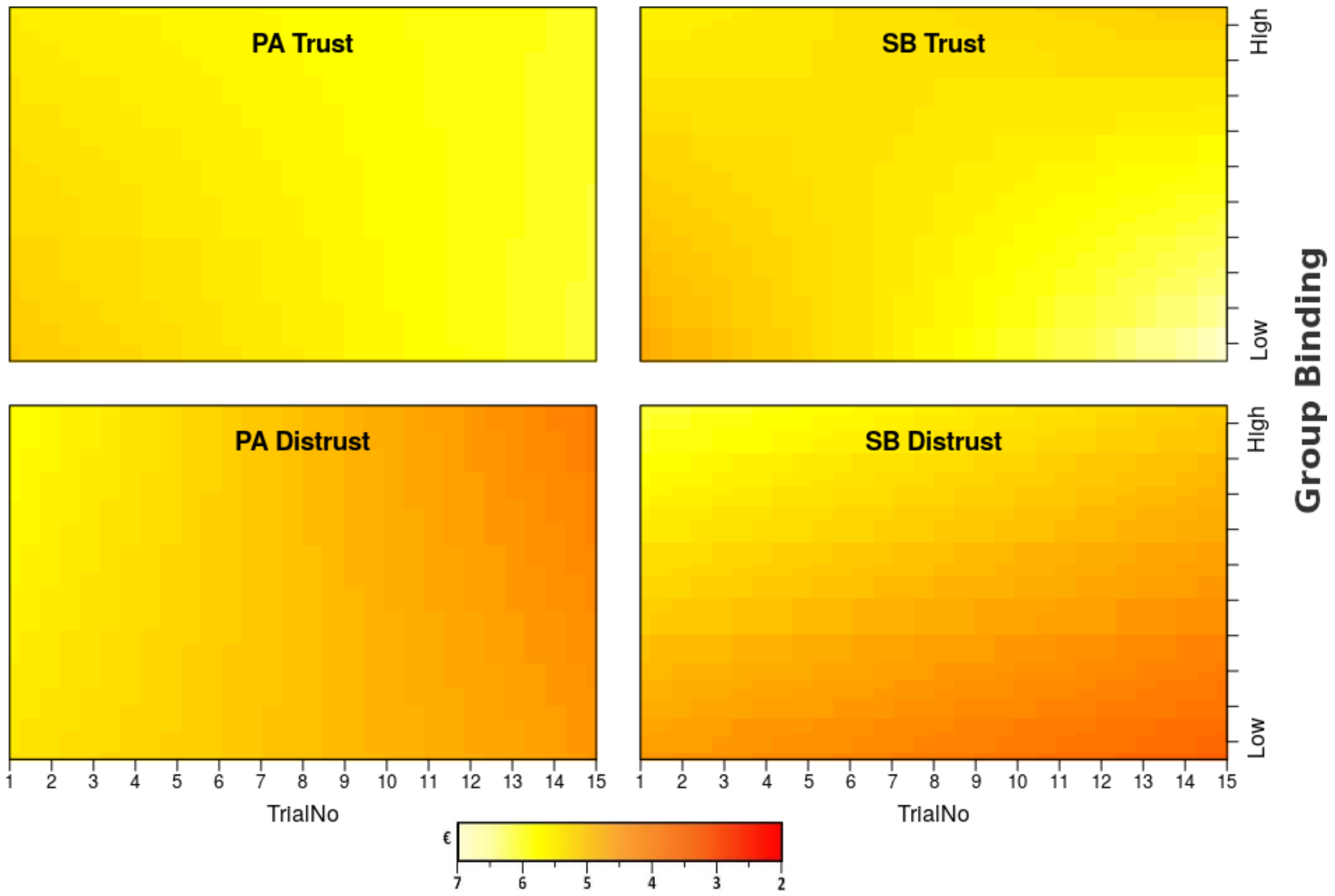

Figure 3. Heat map model of the economic behavior of participants as a function of their scores on the group-binding dimension. The panels on the left indicate participants' economic behavior during the Trust Game with Piero Angela (PA), while those on the right indicate participants' economic behavior throughout the Trust Game with Silvio Berlusconi (SB). The upper panels indicate the change of participants' behavior in response to a trustworthy game partner, while the lower panels display the participants' adjustment to an untrustworthy game partner. The color-grading provides coarse-grained orientation on the probability to invest a certain amount: red color indicates low, while yellow color high probability to invest. The legend provides fine-grained insight into the numerical gradient of change in investment. For instance, a sum of 6 euro or higher is likely to be invested in the very first trials by participants with high scores on group-binding dimension who are playing with Berlusconi (upper-left quadrant of the upper-right panel), but in the second half of the experiment, it steeply increases its likelihood to be invested by participants who score low on the group-biding dimension and are playing with Berlusconi (bottom-right quadrant of the same panel).

Table 1. **Self-reported Ideology as indicator of economic behavior:**
Step-wise regression analysis of models with increasing complexity of interaction.
Models are denoted in R *lme*4 *update* notation. + = $p < .09$; * = $p < .05$; ** = $p < .01$; *** = $p < .001$

No	Formula	Df	AIC	ΔAIC	logLik	deviance	Chisq	Chi Df	Pr(> Chisq)
m00	Offer ∼ (1|ID)	3	8316.4		-4155.2	8310.4			
m01	m00 .∼.+ Condition	4	8305.5	-10.9	-4148.7	8297.5	12.92	1	***
m02	m01 .∼.+ Group	5	8300.4	-16.0	-4145.2	8290.4	7.02	1	**
m03	m02 .∼.+ Condition : Group	6	8300.8	-15.6	-4144.4	8288.8	1.69	1	
m04	m03 .∼.+ Trustee	7	8302.3	-14.1	-4144.1	8288.3	0.47	1	
m05	m04 .∼.+ Condition : Trustee	8	8304.3	-12.1	-4144.1	8288.3	0.02	1	
m06	m05 .∼.+ Group : Trustee	9	8305.8	-10.6	-4143.9	8287.8	0.44	1	
m07	m06 .∼.+ Condition : Group : Trustee	10	8296.4	-20.0	-4138.2	8276.4	11.38	1	***
m08	m07 .∼.+ TrialNo	11	8294.8	-21.6	-4136.4	8272.8	3.64	1	.
m09	m08 .∼.+ TrialNo : Condition	12	8236.8	-79.6	-4106.4	8212.8	60.03	1	***
m10	m09 .∼.+ TrialNo : Trustee	13	8237.5	-78.9	-4105.7	8211.5	1.26	1	
m11	m10 .∼.+ TrialNo : Condition Trustee	14	8238.1	-78.3	-4105.0	8210.1	1.42	1	
m12	m11 .∼.+ TrialNo : Group	15	8238.2	-78.2	-4104.1	8208.2	1.86	1	
m13	m12 .∼.+ TrialNo : Condition: Group	16	8238.9	-77.5	-4103.4	8206.9	1.34	1	
m14	m13 .∼.+ TrialNo : Group : Trustee	17	8240.8	-75.6	-4103.4	8206.8	0.11	1	
**m15**	**m14 .∼.+ TrialNo * Condition * Group * Trustee**	**18**	**8235.8**	**-80.6**	**-4099.9**	**8199.8**	**6.98**	**1**	**

Table 2. **Self-reported Ideology as indicator of economic behavior:**
Descriptive statistics of the winning model Condition*Group*Trustee*TrialNo: Parameter estimates (b), standard errors (SE), lower level (LL) and upper level (UL) estimates for confidence intervals (99% CI). Confidence intervals were computed through profile likelihood. Parameters with CI which do not include zero are highlighted in bold.

	b	SE b	LL	UL
**(Intercept)**	**4.91**	**0.45**	**3.75**	**6.08**
Condition	0.96	0.65	-0.71	2.64
Group	1.17	0.67	-0.57	2.91
Trustee	0.00	0.66	-1.71	1.70
TrialNo	0.08	0.03	-0.01	0.17
Condition : Group	-0.51	0.95	-2.97	1.94
Condition : Trustee	-1.09	0.93	-3.50	1.32
Group : Trustee	-0.51	0.95	-2.96	1.95
**Condition : TrialNo**	**-0.19**	**0.05**	**-0.32**	**-0.07**
Trustee : TrialNo	0.06	0.05	-0.07	0.19
Group : TrialNo	0.00	0.05	-0.14	0.13
Condition : Group : Trustee	1.46	1.33	-1.98	4.89
Condition : Trustee : TrialNo	-0.08	0.07	-0.26	0.11
Condition : Group : TrialNo	-0.08	0.07	-0.27	0.11
Group : Trustee : TrialNo	-0.12	0.07	-0.31	0.07
**Condition : Group : Trustee : TrialNo**	**0.27**	**0.10**	**0.01**	**0.53**

Table 3. **Group binding dimension as indicator of economic behavior:**
Step-wise regression analysis of models with increasing complexity of interaction.
Models are denoted in R *lme*4 *update* notation. + = $p < .09$; * = $p < .05$; ** = $p < .01$; *** = $p < .001$

No	Formula	Df	AIC	ΔAIC	logLik	deviance	Chisq	Chi Df	Pr(> Chisq)
m00	Offer ∼ (1∣ID)	3	8316.4		-4155.2	8310.4			
m01	m00 .∼.+ GroupBind	4	8305.5	-10.9	-4148.7	8297.5	12.92	1	***
m02	m01 .∼.+ Group	5	8305.3	-11.1	-4147.7	8295.3	2.15	1	
m03	m02 .∼.+ GroupBind : Group	6	8305.1	-11.3	-4146.5	8293.1	2.23	1	
m04	m03 .∼.+ Trustee	7	8306.7	-9.7	-4146.3	8292.7	0.39	1	
m05	m04 .∼.+ GroupBind : Trustee	8	8308.7	-7.7	-4146.3	8292.7	0.01	1	
m06	m05 .∼.+ Group : Trustee	9	8309.8	-6.6	-4145.9	8291.8	0.85	1	
m07	m06 .∼.+ GroupBind : Group : Trustee	10	8308.9	-7.5	-4144.4	8288.9	2.93	1	.
m08	m07 .∼.+ TrialNo	11	8307.3	-9.1	-4142.6	8285.3	3.64	1	.
m09	m08 .∼.+ TrialNo : GroupBind	12	8249.2	-67.2	-4112.6	8225.2	60.03	1	***
m10	m09 .∼.+ TrialNo : Trustee	13	8250.0	-66.4	-4112.0	8224.0	1.26	1	
m11	m10 .∼.+ TrialNo : GroupBind Trustee	14	8250.5	-65.9	-4111.3	8222.5	1.42	1	
m12	m11 .∼.+ TrialNo : Group	15	8246.8	-69.6	-4108.4	8216.8	5.76	1	*
m13	m12 .∼.+ TrialNo : GroupBind: Group	16	8247.4	-69.0	-4107.7	8215.4	1.37	1	
m14	m13 .∼.+ TrialNo : Group : Trustee	17	8248.6	-67.8	-4107.3	8214.6	0.75	1	
**m15**	**m14 .∼.+ TrialNo * GroupBind * Group * Trustee**	18	8247.9	-68.5	-4106.0	8211.9	2.72	1	.

Table 4. **Group binding dimension as indicator of economic behavior:**
Descriptive statistics of the winning model Condition*GroupBind*Trustee*TrialNo: Parameter estimates (b), standard errors (SE), lower level (LL) and upper level (UL) estimates for confidence intervals (99% CI). Confidence intervals were computed through profile likelihood. Parameters with CI which do not include zero are highlighted in bold.

	b	SE b	LL	UL
**(Intercept)**	**5.46**	**0.35**	**4.56**	**6.36**
Condition	0.74	0.49	-0.53	2.00
GroupBind	0.26	0.31	-0.54	1.06
Trustee	-0.26	0.49	-1.53	1.02
**TrialNo**	**0.08**	**0.03**	**0.01**	**0.14**
Condition : GroupBind	-0.05	0.48	-1.31	1.20
Condition : Trustee	-0.38	0.69	-2.16	1.40
GroupBind : Trustee	0.3	0.51	-1.03	1.63
**Condition : TrialNo**	**-0.23**	**0.04**	**-0.32**	**-0.14**
Trustee : TrialNo	0.01	0.04	-0.09	0.10
GroupBind : TrialNo	-0.02	0.02	-0.08	0.04
Condition : GroupBind : Trustee	0.28	0.71	-1.55	2.12
Condition : Trustee : TrialNo	0.05	0.05	-0.08	0.18
Condition : GroupBind : TrialNo	-0.01	0.04	-0.10	0.09
GroupBind : Trustee : TrialNo	-0.07	0.04	-0.17	0.03
Condition : GroupBind : Trustee : TrialNo	0.09	0.05	-0.05	0.22

Table 5. Social equality dimension as indicator of economic behavior:
Step-wise regression analysis of models with increasing complexity of interaction.
Models are denoted in R *lme*4 *update* notation. + = $p < .09$; * = $p < .05$; ** = $p < .01$; *** = $p < .001$

No	Formula	Df	AIC	ΔAIC	logLik	deviance	Chisq	Chi Df	Pr(> Chisq)
m00	Offer ∼ (1|ID)	3	8316.4		-4155.2	8310.4			
m01	m00 .∼.+ SocEqual	4	8305.5	-10.9	-4148.7	8297.5	12.92	1	***
m02	m01 .∼.+ Group	5	8307.2	-9.2	-4148.6	8297.2	0.31	1	
m03	m02 .∼.+ SocEqual : Group	6	8306.5	-9.9	-4147.2	8294.5	2.69	1	
m04	m03 .∼.+ Trustee	7	8308.2	-8.2	-4147.1	8294.2	0.28	1	
m05	m04 .∼.+ SocEqual : Trustee	8	8310.2	-6.2	-4147.1	8294.2	0.01	1	
m06	m05 .∼.+ Group : Trustee	9	8311.9	-4.5	-4146.9	8293.9	0.30	1	
m07	m06 .∼.+ SocEqual : Group : Trustee	10	8306.0	-10.4	-4143.0	8286.0	7.84	1	**
m08	m07 .∼.+ TrialNo	11	8304.4	-12.0	-4141.2	8282.4	3.64	1	.
m09	m08 .∼.+ TrialNo : SocEqual	12	8246.4	-70.0	-4111.2	8222.4	60.03	1	***
m10	m09 .∼.+ TrialNo : Trustee	13	8247.1	-69.3	-4110.5	8221.1	1.26	1	
m11	m10 .∼.+ TrialNo : SocEqual Trustee	14	8247.7	-68.7	-4109.8	8219.7	1.42	1	
m12	m11 .∼.+ TrialNo : Group	15	8249.4	-67.0	-4109.7	8219.4	0.28	1	
m13	m12 .∼.+ TrialNo : SocEqual: Group	16	8251.3	-65.1	-4109.6	8219.3	0.14	1	
m14	m13 .∼.+ TrialNo : Group : Trustee	17	8253.1	-63.3	-4109.6	8219.1	0.14	1	
m15	m14 .∼.+ TrialNo * SocEqual * Group * Trustee	18	8254.8	-61.6	-4109.4	8218.8	0.36	1	

Table 6. Social equality dimension as indicator of economic behavior:
Descriptive statistics of the full model Condition*SocEqual*Trustee*TrialNo: Parameter estimates (b), standard errors (SE), lower level (LL) and upper level (UL) estimates for confidence intervals (99% CI). Confidence intervals were computed through profile likelihood. Parameters with CI which do not include zero are highlighted in bold.

	b	SE b	LL	UL
**(Intercept)**	**5.53**	**0.35**	**4.62**	**6.44**
Condition	0.66	0.49	-0.61	1.94
SocEqual	-0.48	0.40	-1.52	0.56
Trustee	-0.27	0.49	-1.54	1.01
**TrialNo**	**0.07**	**0.03**	**0.01**	**0.14**
Condition : SocEqual	0.52	0.53	-0.86	1.89
Condition : Trustee	-0.42	0.69	-2.20	1.36
SocEqual : Trustee	0.68	0.48	-0.55	1.91
**Condition : TrialNo**	**-0.23**	**0.04**	**-0.32**	**-0.13**
Trustee : TrialNo	0.00	0.04	-0.09	0.10
SocEqual : TrialNo	0.03	0.03	-0.05	0.11
**Condition : SocEqual : Trustee**	**-2.05**	**0.78**	**-4.07**	**-0.04**
Condition : Trustee : TrialNo	0.06	0.05	-0.08	0.19
Condition : SocEqual : TrialNo	-0.03	0.04	-0.13	0.07
SocEqual : Trustee : TrialNo	-0.02	0.04	-0.12	0.07
Condition : SocEqual : Trustee : TrialNo	0.04	0.06	-0.12	0.19

Table 7. **2 x 2 ANOVA with the Group (LW/RW) and the Trustee (SB/PA) as between-subjects factor and the moral values or social attitudes as dependent variables**

**Ideology Measures**						
Moral Foundations	***t* (1,119)**	**p-value**	LW		RW	
			Mean	SD	Mean	SD
Harm	1.31	0.192	23.60	2.95	22.78	3.94
Fairness	4.16	$<$ 0.001	25.10	2.94	22.72	3.33
Loyalty	-5.74	$<$ 0.001	19.17	3.74	23.10	3.79
Authority	-6.31	$<$ 0.001	14.81	4.59	20.07	4.58
Purity	-6.44	$<$ 0.001	12.16	4.64	17.41	4.31
SDO	-6.91	$<$ 0.001	2.45	0.77	3.66	1.14
RWA	-11.26	$<$ 0.001	2.56	0.82	4.34	0.91
ESJ	-6.55	$<$ 0.001	3.01	0.73	3.94	0.83
SVO	1.88	0.063	32.33	9.07	28.68	12.13

Table 8. **One-way ANOVA on 121 participants with the Group (RW/LW) as between-subjects factor, and the ratings regarding Piero's Angela Political Orientation as dependent variable**. The groups do not differ in the political categorization of Angela i.e., neither LW-voters nor RW-voters perceived him as more politically inclined toward the liberal/conservative ideologies.

**Political Orientation: Evaluation of Piero Angela**						
	**F(1, 119) = 3.32**	**N°**	**Mean**	**SD**	**+95% CI**	**-95% CI**
**Group**	***p-value***	121	-0.31	0.80	-0.45	-0.17
LW	**.071**	63	-0.44	0.80	-0.64	-0.23
RW		58	-0.17	0.79	-0.38	0.04

Table 9. Repeated measures ANOVA on 121 participants with the Trustee (SB/PA) as within-subjects factor, the Group (RW/LW) as between-subjects factor, and the following personality traits as dependent variables: Competence, Positive and Negative Valence, Dominance and Acquaintance. The groups do not differ in the assessment of Angela on each variable, but they do in the assessment of Berlusconi regarding his Competence, Positive and Negative Valence. Importantly, there is no evident between-group distinction in the ratings for Dominance and Acquaintance of the character, since both groups assign higher scores to Berlusconi on both dimensions.

**Personality Measures**						
Competence<br>Group (LW/RW) x Trustee (PA/SB)	***F*(1,119)**	**p-value**	LW		RW	
	14.15	< 0.001	Mean	SD	Mean	SD
PA		0.286	4.28	0.93	4.46	0.92
SB		< 0.001	2.52	1.25	3.72	1.05
Positive Valence<br>Group (LW/RW) x Trustee (PA/SB)	***F*(1,119)**	**p-value**	LW		RW	
	18.98	< 0.001	Mean	SD	Mean	SD
PA		0.699	3.51	1.06	3.43	1.12
SB		< 0.001	1.81	1.03	2.86	1.19
Negative Valence<br>Group (LW/RW) x Trustee (PA/SB)	***F*(1,119)**	**p-value**	LW		RW	
	33.36	< 0.001	Mean	SD	Mean	SD
PA		0.121	1.57	0.78	1.84	0.89
SB		< 0.001	4.20	1.00	3.09	1.17
Trustee Evaluation: Dominance<br>Group (LW/RW) x Trustee (PA/SB)	***F*(1,119)**	**p-value**	LW		RW	
	0.71	0.402	Mean	SD	Mean	SD
PA		0.884	2.302	0.891	2.328	1.066
SB		0.216	4.222	0.792	4.431	1.045
Trustee Evaluation: Acquaintance<br>Group (LW/RW) x Trustee (PA/SB)	***F*(1,119)**	**p-value**	LW		RW	
	2.52	0.115	Mean	SD	Mean	SD
PA		0.849	2.857	1.105	2.897	1.112
SB		< 0.05	3.762	0.979	4.190	1.165
